# A modified culture medium for improved isolation of marine vibrios

**DOI:** 10.1002/mbo3.835

**Published:** 2019-07-18

**Authors:** Marcello Tagliavia, Monica Salamone, Carmelo Bennici, Paola Quatrini, Angela Cuttitta

**Affiliations:** ^1^ IAS‐CNR Campobello di Mazara Italy; ^2^ Department of Biological, Chemical and Pharmaceutical Sciences and Technologies (STEBICEF) University of Palermo Palermo Italy; ^3^ Abiel s.r.l Campobello di Mazara Italy

**Keywords:** marine *Vibrio*, modified TCBS, *Vibrio* isolation and enumeration, *Vibrio*‐specific PCR primers

## Abstract

Marine *Vibrio* members are of great interest for both ecological and biotechnological research, which often relies on their isolation. Whereas many efforts have been made for the detection of food‐borne pathogenic species, much less is known about the performances of standard culture media toward environmental vibrios. We show that the isolation/enumeration of marine vibrios using thiosulfate‐citrate‐bile salts‐sucrose agar (TCBS) as selective medium may be hampered by the variable adaptability of different taxa to the medium, which may result even in isolation failure and/or in substantial total count underestimation. We propose a modified TCBS as isolation medium, adjusted for marine vibrios requirements, which greatly improved their recovery in dilution plate counts, compared with the standard medium. The modified medium offers substantial advantages over TCBS, providing more accurate and likely estimations of the actual presence of vibrios. Modified TCBS allowed the recovery of otherwise undetected vibrios, some of which producing biotechnologically valuable enzymes, thus expanding the isolation power toward potentially new enzyme‐producers *Vibrio* taxa. Moreover, we report a newly designed *Vibrio*‐specific PCR primers pair, targeting a unique *rpoD* sequence, useful for rapid confirmation of isolates as *Vibrio* members and subsequent genetic analyses.

## 1 INTRODUCTION

Microbial enzymes have wide applications in several fields, ranging from food manufacturing/processing to biomedical‐pharmaceutical applications. Selection of microorganisms for enzyme production at the industrial level requires good yield and high production rate of active biomolecules. In this scenario, marine enzymes are considered among the most promising; some are tolerant to a wide range of pH, temperature, and other harsh conditions required in industrial and biomedical applications. Novelty in their structure and characteristics has shown promising scope to the researchers in academia and industry (Rao, Imchen, & Kumavath, 2017).

Therefore, enzymes produced by marine microorganisms often show higher stability and/or activity in operational conditions (i.e., relatively high ionic strength, low or high temperature, extreme pH values) and different types of functional properties when compared with enzymes isolated by conventional sources (Burton, Cowan, & Woodley, 2002; Ferrer, Martinez‐Abarca, & Golyshin, 2005; Trincone, 2011; Dionisi, Lozada, & Olivera, 2012).

Moreover, the use of seawater as reaction medium has been suggested as an interesting alternative for specific processes (Domínguez de María, 2013), so that marine enzymes may represent ideal biocatalysts for such applications, as well as for those where their use could be considered more obvious, that is, processing of wastes of marine origin, like fishes/seafood manufacturing wastes, the latter being a promising source of valuable bioproducts (Ishak & Sarbon, 2018; Sánchez & Vázquez, 2017).

The genomic and metabolic diversity of prokaryotes is an extraordinary—still relatively underexploited—source of new products, including enzymes. In this context, even if extremophiles are usually considered as the most promising and underexplored source of enzymes of biotechnological interest (Zhang & Kim, 2010), members of other much more studied genera may still represent a valuable, sometimes underestimated, source of very useful enzymes.

In the era of metagenomics, which has emerged as a strategic approach to explore the huge heterogeneity of environmental bacteria (most of which, more than 99%, are unculturable) through the sequencing and analysis of DNA extracted from environmental samples, as well as through functional screening of metagenomics libraries, many efforts of mining functions are focused on such approaches. However, such strategies, despite their enormous potential, are not without limitations.

In fact, the enzyme screening approach involves the heterologous expression of proteins encoded by environmental DNA in a surrogate host (usually *Escherichia coli*), followed by the metagenomic libraries screening, which is carried out by assays for enzymatic activity against a specific substrate. Such approach allows mining for new enzymes, and discovery of novel enzyme families (with no sequence similarity to previously characterized proteins) (Popovic et al., 2017).

However, the entire workflow as well the correct assessment of enzymatic activity, may be hampered by the well‐known limitations associated with the heterologous expression. In this scenario, the screening of culturable marine bacteria for enzymatic activities should still be considered as a valid approach, which offers obvious advantages.

In the context of mining proteolytic activities for biotechnological purposes, the members of genus *Vibrio* are among the most promising, as well‐known proteases‐producing bacteria. Moreover, as aquatic bacteria, they are expected to produce highly active secreted enzymes, able to recognize and digest even very diluted substrates. They also have the ability to move small molecules into the periplasmic space where digestion can occur.

Many proteolytic enzymes are secreted by several bacteria in order to convert high molecular weight polypeptides into shorter chains, for an easier uptake and utilization, as well as virulence factors (in pathogenic vibrios). Such enzymes are of high interest for biotechnological applications (Salamone et al., 2015 and references therein); in particular, proteolytic enzymes from marine organisms are particularly attractive and promising for biomedical applications such as tissue dissociation and cell isolation for transplantation/cell‐therapy purposes (Brandhorst, Brandhorst, & Johnson, 2017; Miyoshi, 2013 ), so that the screening of environmental bacteria for such activities can lead to the discovery of new suitable enzymes.

Thus, the extensive screening of environmental *Vibrio* isolates is still worthy as a promising strategy; in fact, its power relies on the maximum recovery of the *Vibrio* diversity originally present in the natural sample.

Moreover, besides their biotechnological potential, including biomedical applications (Salamone et al., 2015), the interest toward the genus *Vibrio* (not only pathogenic species) in marine environments has increased in last years, due not only to their ecological role in natural ecosystems (Herbert, 1999; Zhang et al., 2015) but also to their dynamics in aquatic environments in response to climate changes (Narracci, Acquaviva, & Cavallo, 2014; Vezzulli et al., 2012, 2016).

*Vibrio* is a bacterial genus well known for its high frequency of gene exchange (especially some clades), leading to a very rapid evolution and genomic plasticity; thus, strains with novel arrays of functions, as well as—presumably—physiological features (including the ability to grow on specific culture media), are expected to arise continuously. Such features make them sometimes difficult to be assigned to species (Ke et al., 2017; Urbanczyk, Ogura, & Hayashi, 2014), but also of high interest, even in the perspective of biotechnological applications.

Various selective and/or differential culture media are employed to isolate *Vibrio* species from environmental samples, most of which aim to detect pathogenic vibrios (Froelich, Weiss, & Noble, 2014; Williams, Froelich, & Oliver, 2013, Griffitt & Grimes, 2013 and references therein). Some of these media allow the growth of selected *Vibrio* species only (Oliver, 2006).

Thiosulfate‐citrate‐bile salts‐sucrose agar was one of the first selective media used for the isolation and purification of vibrios (Oliver, 2006), and is still largely employed. TCBS has been widely employed for the isolation of pathogenic vibrios (*V*. *cholerae*, *V. parahaemolyticus*, *V. vulnificus*) from clinical specimens and food, as well as from the aquatic environment. Moreover, it is also indicated as isolation/enumeration medium for all vibrios (except *V. hollisae *and *V. metschnikovii*), a bacterial group abundant mainly in estuarine and sea waters, as well as seafood (Madigan et al., 2014).

On this medium, different *Vibrio* species (i.e., *V. cholerae*, *V. alginolyticus*, *V. harveyi*) can be differentiated based on the ability of fermenting sucrose, which results in yellow colonies; in contrast, sucrose nonfermenters, such as *V. vulnificus* and *V. parahaemolyticus*, appear in green colonies (Thompson, Iida, & Swings, 2004). Despite the selectivity for vibrios, other genera such as *Staphylococcus*, *Flavobacterium*, *Pseudoalteromonas*, and *Shewanella* can grow on TCBS as well (Thompson et al., 2004), but their colonies can be differentiated from *Vibrio* ones due to their distinctive phenotypes.

For marine vibrios, similarly to most marine bacteria, a medium salt composition which could resemble that found in sea water is expected to be beneficial; nevertheless, TCBS agar salt content is very different and likely poorly suitable to ensure the maximum recovery of marine vibrios, just because it is not specifically intended for their isolation from marine environments. Thus, biases in isolation and quantification of this bacterial group in such samples might be suspected. This concern has not been investigated nor reported to date.

In this study, we show that TCBS medium cannot be actually considered fully suitable for marine vibrios isolation, whose recovery was substantially improved through some medium modifications, thus improving environmental surveys aiming to isolate biotechnologically valuable enzyme‐producer vibrios. In particular, we verified that salt composition of TCBS could hamper the recovery of marine vibrios. We tested various modifications of the medium in order to overcome such limit, so as to ensure the maximum recovery of marine vibrios onto agar plates. The correction of the salt composition in standard TCBS leads to a substantial improvement of total *Vibrio* counts from marine specimens, as demonstrated by the recovery of isolates unable to grow onto standard TCBS medium. Moreover, a *Vibrio*‐specific primers pair, targeting a unique *rpoD* sequence, was designed in order to quickly further confirm isolates as *Vibrio* members.

## 2 MATERIALS AND METHODS

### 2.1 Sampling

Samples (*Muraena helena*) were recovered from local fisheries, just after catching (at maximum depth of 10 m). Microbiological samplings were performed immediately or within a few hours after sampling. In particular, buccal and skin swabs (1 cm^2^ of sterile filter paper) from 10 individuals, were dissolved into sterile sea water, serially diluted and plated onto selected culture media.

### 2.2 Microbiological methods

#### 2.2.1 Culture media

Marine Broth (MB, Laboratorios CONDA, Spain) and Marine Agar (MB containing 1.5% Agar) were used as nonselective media. TCBS (Laboratorios CONDA, Spain) was prepared following the manufacturer's instructions for the standard medium. mTCBS and mTCBS‐2 were prepared by dissolving the powder in 100% and 75% filter‐sterilized sea water (sea water:distilled water 3:1), respectively; mTCBS‐aSW was prepared using artificial SW (containing 30 g/L of marine coral reef aquarium salts); TCBS‐S consisted of the standard medium, supplemented with NaCl 27 g/L; TCBS + MB consisted in TCBS dissolved in MB.

#### 2.2.2 Bacterial counts and isolation

After dilution, aliquots of 0.2 ml were plated onto each medium and incubated at 24°C for 18–24 hr. All tests were carried out at least in triplicate.

For isolation, single colonies were picked and stroked onto new plates. Liquid cultures were incubated overnight at 24°C in an orbital shaker.

#### 2.2.3 Type strains

*Vibrio* type strains were from DSMZ (Germany), and in particular *V. parahaemolyticus *(DSM10027), *V. vulnificus *(DSM10143), *V. alginolyticus *(DSM2171), *V. owensii *(DSM2165), *V. campbellii *(DSM19270), *V. harveyi *(DSM19623), *V. splendidus *(DSM19640). Moreover, several environmental isolates, previously identified as *Vibrio *spp. (most of which assigned to the *V. harveyi* Clade) were tested as well. Similarly, *Shewanella *spp., *Pseudoalteromonas *spp., *Cellulophaga lytica,* and *Ruegeria *spp*.* were used in order to confirm the selectivity of modified media.

#### 2.2.4 Bacterial identification

Cells from single colonies or small amounts of pellets from pure liquid cultures were processed by a fast lysis protocol (Tagliavia et al., 2016) and used as template in PCR. All reactions were performed in iCycler thermal cycler (BioRad), a final volume of 25 µl containing 1X Phire HSII DNA Polymerase Buffer (Thermoscientific), primers 0.5 µM each, dNTPs 0.2 mM each, 0.25 µl Phire HSII DNA Polymerase, 1 Phire HSII DNA Polymerase, 1 µl cell lysate, using cycling conditions suggested by enzyme manufacturer. PCR primers are listed in Table 2.

Amplicons were sequenced by Sanger method by Macrogen Europe (The Netherlands).

Sequences were analyzed by BLAST for the best match.

#### 2.2.5 Primers design

*Vibrio*‐specific *rpoD* primers, V.rpoD66f and V.rpoD1592r (expected amplicon size of 1500bp), were designed based on conserved regions present exclusively in *Vibrio*‐*rpoD* gene, identified by sequence alignment by ClustalOmega (http://www.ebi.ac.uk/Tools/msa/clustalo/) of *rpoD* sequences available in GenBank: *V. campbellii *(sequence ID:CP006605.1), *V. harveyi* (Sequence ID: CP009467.2), *V. parahaemolyticus* (Sequence ID: CP006008.1), *V. alginolyticus *(Sequence ID: CP006718.1), *V. vulnificus *(Sequence ID: AE016795.3), *V. tubiashii *(Sequence ID: CP009354.1), *V. splendidus *(Sequence ID: FM954972.2), *V. anguillarum *(Sequence ID: CP011436.1), *V. coralliilyticus *(Sequence ID: CP009264.2), *V. cholerae* *O395* (Sequence ID: CP001235.1), *V. cholerae* *O1 *(Sequence ID: KM660639.1).

Sequences and primers were first challenged with bacterial sequences by BLAST analysis, in order to check their uniqueness and specificity. Then, primers were tested in vitro using *Vibrio* type strains (DSMZ, see above), *Vibrio *isolates, and other bacteria, namely *E. coli *(MG1655), *Shewanella *spp., *Pseudoalteromonas *spp., *Cellulophaga lytica*, and *Ruegeria *spp.

For DNA sequencing, forward primers bearing the M13F sequence (5′‐TTGTAAAACGACGGCCAGT‐3′) added at their 5′ end, as indicated in the primers table (Table 2) were used, and the M13F employed as sequencing primer.

#### 2.2.6 SDS electrophoresis and zymography

Aliquots of overnight bacterial cultures in MB were cleared by centrifugation and fractionated by sodium dodecyl sulfate‐polyacrylamide gel electrophoresis (SDS‐PAGE), carried out as described by Laemmly, 1970. After electrophoresis, the gels were stained with 0.25% Coomassie Brilliant Blue G‐250. Zymography was performed on native‐PAGE (Kin et al., 2007). After electrophoresis, gelatin zymographies were incubated for 24 hr at 37°C in two developing buffers: Activator buffer containing 2 mmol/L CaCl_2_, Tris‐HCl buffer (50 mmol/L; pH 7.4), containing 1.5% Triton X‐100 and 0.02% NaN_3_ plus inhibitor buffer Tris‐HCl buffer (50 mmol/L; pH 7.4), containing 1.5% Triton X‐100 and 0.02% NaN_3 _plus 2 mmol/L EDTA to inhibit any gelatinase activity. After incubation, gels were stained using Coomassie Brilliant Blue G‐250.

## 3 RESULTS

### 3.1 Tests on modified culture media

The oral cavity of the mediterranean moray eel (*Muraena helena*) was chosen as a possible source of diverse *Vibrio *isolates because of the feeding behavior of such fish species and of the unique anatomical features of its mouth. In fact, debris from preys usually remain trapped between teeth, making that environment nutrient‐rich especially for bacterial species able to efficiently degrade tissues components. Thus, vibrios were expected to be well represented as commensals in the oral microbial communities. Moreover, a protease‐producing strain, identified as *Vibrio parahaemolyticus*, had been previously isolated from a similar sample and its enzymes were characterized (Salamone et al., 2015). Such considerations prompted us to consider moray eel microbial communities as a promising source of proteases‐producing isolates, thus worthy of further investigations.

With the aim of improving marine *Vibrio *recovery and isolation, attempts were done in order to assess if the colonies yield onto TCBS agar plates could be increased.

In particular, even if species able to grow on TCBS are well known, it could not be excluded that the isolation of some marine vibrios could be hampered. In particular, we hypothesized that salt composition and/or ionic strength of TCBS, which is very different from that of other isolation media used for marine bacteria cultivation, could hamper the recovery of vibrios from marine samples; thus, a series of TCBS modifications was challenged with natural samples.

In particular, the *Vibrio* colony yield of TCBS (standard medium) was compared with that of various modified media, namely mTCBS, mTCBS‐2, mTCBS‐aSW, TCBS‐S, and TCBS + MB (see Materials and Methods for compositions), all sharing an increased ionic strength compared with TCBS.

**Figure 1:**
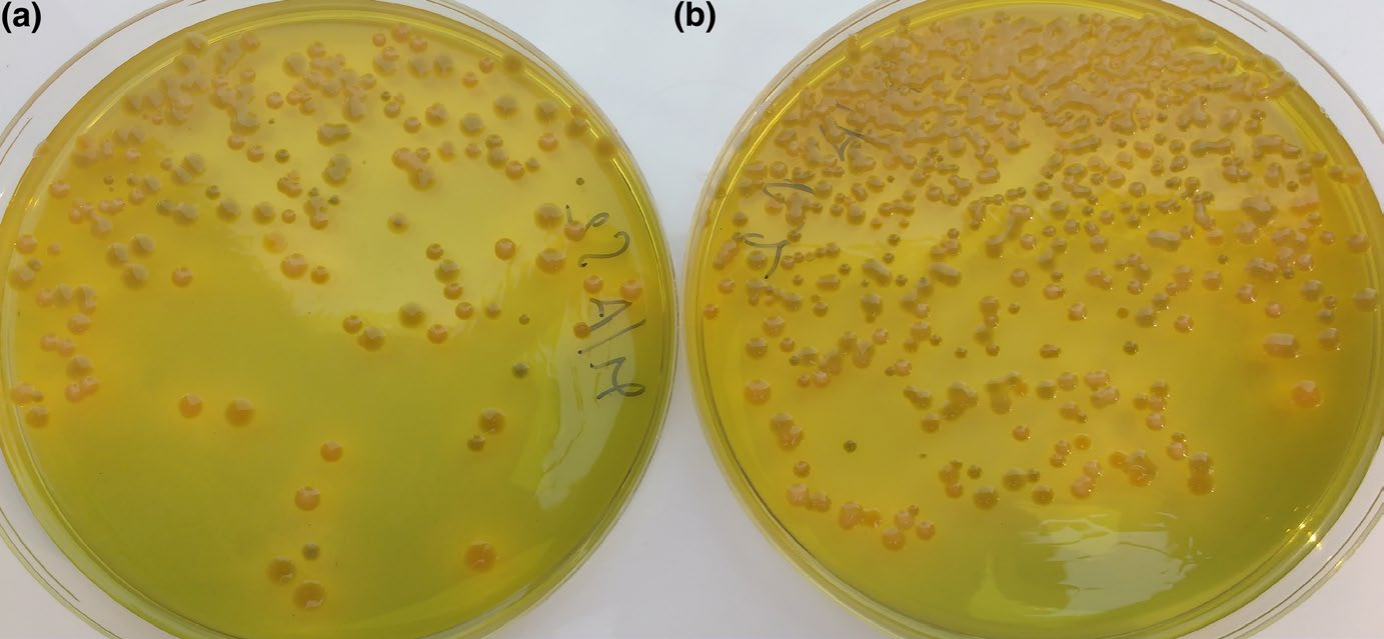
Improved plate count on mTCBS. Comparison of *Vibrio *spp. plate counts of a dilution from a fish buccal swab. (a) Colonies on thiosulfate‐citrate‐bile salts‐sucrose agar (TCBS) (standard medium). (b) Colonies onto mTCBS (modified medium)

**Figure 2:**
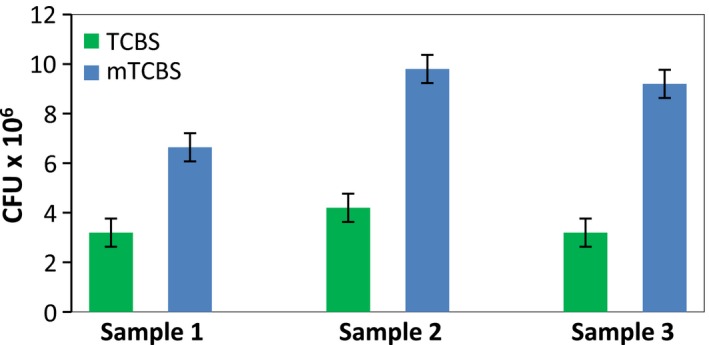
Comparison of *Vibrio* recovery with different media. *Vibrio* spp. colony yield from three randomly selected fish buccal swabs plated on TCBS and mTCBS

Using all modification but TCBS‐S, an average of twofold increase in the number of *Vibrio* colonies was observed, compared to standard medium (Figures 1 and 2). The supplementation of TCBS with NaCl did not result in the increased count obtained with all other modifications, thus suggesting that the improved recovery achieved with other formulations results mainly from the overall salt content (i.e., the availability of a mixture of chemical elements), rather than from the ionic strength itself.

A slight reduction of salt content, using 75% sea water (mTCBS) or a SW (mTCBS‐aSW) instead of pure SW (as in TCBS‐2), proved to be beneficial, as it improved the colony size without reducing the advantage of modified medium over standard TCBS, presumably because of a more balanced overall salt concentration. TCBS + MB supported the most luxuriant growth, resulting in very large colonies, presumably given the much higher nutrient concentration, combined with a balanced salts mixture (data not shown). However, as the possibility—although unlikely—that the combination of the two media could partially impair selectivity could not be fully excluded, for following analyses mTCBS or mTCBS‐aSW were chosen.

In order to verify that salt content modifications did not affect the selectivity, modified media were challenged with known species. All formulations supported the growth of all marine *Vibrio* tested, including type strains, namely *V. parahaemolyticus*, *V. vulnificus*, *V. alginolyticus*, *V. owensii*, *V. campbellii*, *V. harveyi*, *V. splendidus*, along with environmental isolates previously identified as *Vibrio *spp., whereas the growth of bacteria belonging to other genera was inhibited, as expected (Table 1).

**Table 1:** Selectivity of modified media

Strain	TCBS	TCBS‐S	mTCBS	mTCBS‐2	mTCBS‐aSW	TCBS + MB
*V. parahaemolyticus*	+	+	+	+	+	+
*V. vulnificus*	+	+	+	+	+	+
*V. alginolyticus*	+	+	+	+	+	+
*V. owensii*	+	+	+	+	+	+
*V. campbellii*	+	+	+	+	+	+
*V. harveyi*	+	+	+	+	+	+
*V. splendidus*	+	+	+	+	+	+
*Vibrio *sp. (*harveyi *Clade)	+	+	+	+	+	+
Isolate VA	−	−	+	+	+	+
Isolate VD	−	−	+	+	+	+
Isolate VL	−	−	+	+	+	+
Isolate VN	−	−	+	+	+	+
Isolate VD	−	−	+	+	+	+
*Shewanella *spp.	−	−	−	−	−	−
*Pseudoalteromonas *spp.	−	−	−	−	−	−
*Cellulophaga lytica*	−	−	−	−	−	−
*Ruegeria *spp.	−	−	−	−	−	−

TCBS: thiosulfate‐citrate‐bile salts‐sucrose agar.

Colonies obtained onto modified media plates were confirmed as *Vibrio* members by a PCR test (data not shown) using a specifically designed *Vibrio*‐specific *rpoD* primers pair, previously validated both in silico and in vitro (Table 2; see Materials and Methods for further details).

**Table 2:** PCR primers list

Target	Primer name	Sequence (5′‐3′)	Reference
ITS	ITSF	*TCGTAACAAGGTAGCCGTA*	Cardinale et al., 2004
	ITSReub	*GCCAAGGCATCCACC*	
16S rDNA	27f	*M13‐AGAGTTTGATCMTGGCTCAG*	Lane, 1991
	1492r	*TACGGYTACCTTGTTACGACTT*	
VgyrB (*Vibrio*)	VgyrB274F	*M13‐GAAGTTATCATGACGGTACTTC*	Sawabe, Kita‐Tsukamoto, & Thompson, 2007
	VgyrB1171R	*CCTTTACGACGAGTCATTTC*	
VrpoD (*Vibrio*)	V.rpoD66f	*M13‐GACSTACGCMGAAGTAAAYGACCAC*	This work
	V.rpoD1592r	*AGATGCGAATCTTCRTCRTCACC*	

ITS: intergenic spacer.

### 3.2 Modified media improves cells adaption and survival

The results obtained from comparison of standard and modified culture media prompted us to investigate which vibrios could fail to grow onto the standard medium. For this purpose, agar plates from samples which showed the most relevant count differences were chosen for further investigations. In particular, 90 single colonies grown on mTCBS were picked and stroked on both TCBS and mTCBS, in order to identify the bacteria responsible for such plate‐count difference observed. Unexpectedly, 87 of 90 selected isolates grew on both TCBS and mTCBS, whereas only about 50% of them (corresponding approximatively to the ratio CFU_TCBS_/CFU_mTCBS_) were expected to grow. It was hypothesized that the lower colony recovery observed when analyzing natural samples on TCBS could reflect the poor adaptability of most cells of some isolates to the selective medium, rather than the complete inability to grow.

To investigate this possibility, the 87 isolates were grown in marine broth (as nonselective medium), properly diluted, and spotted onto marine agar (MA, as positive control), TCBS and mTCBS plates.

Onto MA and mTCBS, comparable growth was observed with all spots showing confluent growth, while on TCBS 57 of 90 of tested isolates yielded only a few colonies of heterogeneous size (most of which very small) at the lowest dilution and no colonies at the higher ones, thus supporting our hypothesis.

In order to exclude that such results could be due to genetic heterogeneity, both small and large colonies obtained from spots onto TCBS were isolated, grown in MB (to rule out any selection), and tested again. The colony size proven to be not an inherent feature, since normal and even‐sized colonies were obtained on mTCBS and MA, regardless of the size of the starting colony; however, low recovery and colony size heterogeneity were observed again on TCBS. These results supported the hypothesis of the poor adaption rate of cells to TCBS, suggesting that the reduced colony size observed onto TCBS plates might correspond to the late adaption of a minority of single cells to the medium, which results in difficult/delayed growth. Such observations, considered all together, could explain the different recovery power of mTCBS compared to TCBS.

### 3.3 *Vibrio* identification

All *Vibrio* isolates showing reduced or no growth onto TCBS were grouped based on ribosomal intergenic spacer pattern (data not shown), and one isolate representative of each (four in total; namely VD, VL, VN, VO) was selected for molecular identification by 16S rDNA PCR amplification and sequencing.

BLAST analysis assigned the VD and VN sequences to *Vibrio gigantis* and *Vibrio crassostreae* with identical scores; VN and VO were assigned to *Vibrio kanaloae*.

In order to achieve a more precise identification of isolates, two additional taxonomically informative *loci*, namely *rpoD* and *gyrB*, were analyzed both as single sequences and by multi‐locus sequence analysis (MLSA) (Pascual, Macián, Arahal, Garay, & Pujalte, 2010).

BLAST analysis of all *rpoD* sequences, carried out on the region commonly employed for *Vibrio* identification, showed the highest identity (99%) with *Vibrio toranzoniae*; VO sequence showed the same identity with *V. kanaloae*, as well. The analysis of the whole sequence, instead, showed a 94% of identity with *V. crassostreae* for all four sequences; the same matching was found with *V. alginolyticus *and *V. splendidus*, as well, with very similar scores. Instead, MLSA assigned all isolates to *V. crassostreae*. Such different results were probably due to sequences available in databases, in particular fully sequenced genomes (MLSA, in fact, relies on sequenced genomes) present only for a few species, as many sequences found in databases derive from partial sequencing of specific loci, so that the bioinformatics analysis of more extended sequences may result in bias toward species with sequenced genomes.

However, the possibility of horizontal gene transfer between related species, resulting in “admixed” genomes, has not to be excluded, and it might be consistent with the behavior of such isolates.

### 3.4 Proteolytic activities of isolates

**Figure 3:**
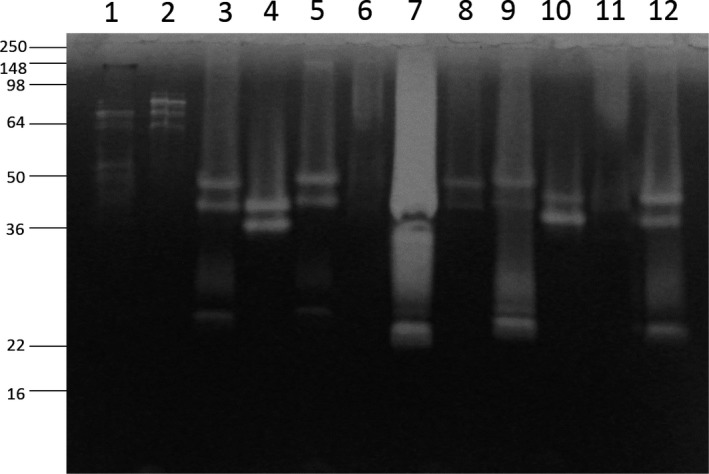
Secreted proteases activity of isolates. Aliquots of culture medium after overnight growth of representative isolates were analyzed to detect gelatinolytic activity of secreted proteases. On the left, positions of molecular weight marker bands are reported. Lane 1–12: *Vibrio* isolates from mTCBS

In order to detect and characterize the proteolytic activity of environmental isolates, including the molecular size distribution, a screening for gelatinase activity after electrophoretic separation was carried out on identified bacteria by zymography. All isolates, including the four characterized ones, showed different patterns of secrete proteolytic enzymes, enriched in molecular weights below 40 KDa (Figure 3). These results suggested their applicative potential as a source of low molecular weight‐secreted proteases, which further functional analyses showed to be largely serine proteases and metalloproteases highly active even in harsh conditions and low temperatures (data not shown).

Low molecular weight enzymes, as well as those which exhibit high stability and activity in extreme conditions, are particularly attractive for their potential biotechnological applications. Indeed, uses where difficult substrates have to be digested (i.e., proteinaceous wastes), or where tight control of low temperatures is a mandatory requirement, may take advantage of such enzyme features. Moreover, small size improves a faster diffusion into tridimensional structures, so that some of the enzymes produced by such isolates are being tested in tissues dissociation. Moreover, such features make such proteases attractive for the ease recombinant and large‐scale production.

## 4 DISCUSSION

Data herein reported clearly indicate that some marine vibrios are unable to grow on selective culture media such as standard TCBS agar, and that they require adjusted medium composition for a successful isolation. In fact, the use of a modified TCBS medium greatly improved the overall recovery of such microorganisms, which resulted in a substantial plate counts increase.

This highlights for the first time, to our knowledge, that vibrios plate count and/or isolation attempts carried out with the standard medium could cause many of them to fail to grow, which leads to an overall underestimation, as well to the loss of bacteria potentially interesting for future exploiting attempts. In particular, some species were found to be mainly responsible of such phenomenon, due to their poor adaption to the standard culture medium.

The use of modified medium allowed us to isolate otherwise undetected vibrios which proved to produce biotechnologically attractive, low molecular weight enzymes, which highlights how improving the isolation power could greatly contribute in expanding the repertoire of suitable enzyme retrieved from natural environments. These data lead us to strongly suggest the use of modified TCBS in any isolation/enumeration effort of marine vibrios.

Noteworthy, isolates able to grow only onto modified TCBS, even if identified as closely related to species not known for their inability to grow onto TCBS, harbored sequences which could be assigned to different species, which made the MLSA not fully reliable. This highlights the extreme plasticity and heterogeneity of *Vibrio* members, thus confirming how much unreliable could be their exact assignation to single species, as pointed out by several authors (Ke et al., 2017; Sawabe et al., 2013; Steinuma et al., 2016; Urbanczyk et al., 2014).

Conversely, such plasticity has to be expected to potentially and continuously generate microorganisms harboring new features (including enzyme production), as well as unpredictable requirements for proper growth in laboratory conditions, which makes them worthy of new screening and isolation efforts aiming to mine new active biomolecules.

The use of improved culture media would be useful in environmental studies, as well. In fact, the interest toward the genus *Vibrio* (not only pathogenic species) in marine environments is being increased in last years, due not only to their ecological role and impact on natural ecosystems (Herbert, 1999; Zhang et al., 2015), but also to their dynamics in aquatic environments in response to climate changes (Vezzulli et al., 2012, 2016). Thus, reliable culture media with the maximum recovery efficiency are useful to assess the presence of vibrios by cultivation, as well as for their isolation.

In addition, the new *Vibrio*‐specific *rpoD* primers pair, targeting a housekeeping gene often employed for *Vibrio *species assessment by DNA sequencing, provides a useful tool for rapid further confirmation of isolates as *Vibrio* genus members by direct PCR, while *rpoD* amplicon sequencing may provide additional information for subsequent species discrimination and identification, compared with sequencing of much shorter fragments (Pascual et al., 2010, and references therein).

Our data demonstrate that the modified isolation media herein proposed greatly improve the recovery and isolation of vibrios from marine samples, for both biotechnological and environmental monitoring purposes. This highlights that the use of adjusted culture media, which could better meet bacterial physiological requirements, should be considered. Moreover, the newly designed *rpoD* primers provided for the easy assessment of the *Vibrio* membership of any isolate and may constitute an improved tool for both identification and taxonomical investigations of *Vibrio* isolates.

## CONFLICT OF INTERESTS

None declared.

## AUTHOR CONTRIBUTIONS

M.T. and P.Q. conceived and designed experiments, and contributed to the writing of the manuscript; M.T. and M.S. conducted experiments; C.B. contributed in conceiving the idea and supported the sampling; A.C. funded the work.

## ETHICS STATEMENT

None required.

## Data Availability

All data are provided in full in the results section of this paper apart from the DNA sequences, which are available at www.ncbi.nlm.nih.gov/gen-bank/ under accession numbers MH671111‐MH671114, MH790289‐MH790292.
